# Generational changes in multiple sclerosis phenotype in North African immigrants in France: A population-based observational study

**DOI:** 10.1371/journal.pone.0194115

**Published:** 2018-03-27

**Authors:** Clotilde Nardin, Clotilde Latarche, Marc Soudant, Camille Dahan, Maud Michaud, Sophie Pittion-Vouyovitch, Francis Guillemin, Marc Debouverie, Guillaume Mathey

**Affiliations:** 1 Nancy University Hospital, Department of Neurology, Nancy, France; 2 Saint-Denis Hospital, Department of Neurology, Saint-Denis, France; 3 Nancy University Hospital, Université de Lorraine, Inserm CIC 1433 Clinical epidemiology, Nancy, France; 4 Université de Lorraine, APEMAC, Nancy, France; Karolinska Institutet, SWEDEN

## Abstract

**Background:**

The incidence of multiple sclerosis (MS) changes from generation to generation in ethnically different immigrants compared with native-born people. We aimed to determine whether there are generational changes in MS phenotypes among North African immigrants in France.

**Methods:**

Cohort study with data from a population-based MS registry to compare the clinical characteristics of 80 first (NAG1) and 167 second (NAG2) generation North Africans with MS living in France with 5200 native-born Europeans. Adjusted Cox models were used to test the association between scores of 3 and 6 on the expanded disability status scale (EDSS) and the “origin/generation” variable.

**Results:**

Cox models for EDSS scores 3 and 6 showed a higher risk of score 3 (hazard ratio = 1.738, 95% confidence interval 1.237 to 2.444; *P* = .002) and 6 (hazard ratio = 2.372, 95% confidence interval 1.626 to 3.462; *P*<.0001) for NAG1 than Europeans. Being NAG2 was not significantly associated with higher hazards of scores 3 and 6.

**Conclusions:**

We found two different phenotypes among NAG1 and NAG2 MS patients in France. NAG1, but not NAG2, have a higher risk of disability than Europeans. This raises the question of environmental factors in MS expression, and advocates appropriate patient management according to generation in immigrants.

## Introduction

Most epidemiological studies in multiple sclerosis (MS) published to date concern patients of Caucasian origin living in North America or Europe. The incidence of MS in non-Western countries has been documented [1–4], but data about MS expression are scarce. The expression, or “phenotype” of MS may be assessed by the risk of reaching levels of irreversible disability as assessed by the expanded disability status scale (EDSS) [5]. Some studies have suggested that different phenotypes of MS exist and depend on the ethnic origin of patients in a given country [6–9]. Furthermore, studies of migration between countries where inhabitants have different ethnical origins and environmental exposures and where MS incidences are different show that immigrants tend to acquire the risk of the host country. This is especially true when immigrants are exposed to the host environment in childhood, i.e. if they migrate before the age of 15 [10–12]. A study about patients living in Norway found a lower incidence in the first generation of immigrants from Pakistan compared with their descendants born in Norway—the “second generation”–whose incidence rate was very similar to the population of Norwegian origin [13]. This “generational effect” suggests that early exposure to an environment plays a role in the risk of MS.

France received immigrants in successive waves during the 19^th^ and 20^th^ centuries. Today, 7.8% of the population of Lorraine (a region of Eastern France) originates from North Africa, a quarter of whom were born in their home country [14]. A few studies about MS and the migration of North Africans (NA) have identified a different phenotype of the disease in NA patients compared with Europeans [15,16].

We sought to determine whether the generational effect applies to the MS phenotype in terms of time to irreversible disability, as measured by the EDSS, in first generation (NAG1 and second generation (NAG2) North Africans.

## Materials and methods

Patients were identified through the ReLSEP (*Registre Lorrain des Scléroses En Plaques*) [17], an exhaustive certified registry for MS patients, using data from multiple sources: neurologists, rehabilitation centers, the Health Insurance systems and the French Hospital Information System database. The data for incident cases from 1996 were acquired prospectively (and partly restrospectively for prevalent cases at this date), anonymized and entered in the European Database for Multiple Sclerosis (EDMUS) system [18]. Data collection was approved by the French National Commission for Data Protection and Liberties (CNIL n° 8493536 and 8493536 bis), and confidentiality and safety of data were ensured in accordance with their recommendations. All patients gave their informed consent for their data to be stored in the database and to be used for research purposes.

### Definition of cases and assessment of patients

Diagnosis of MS was established according to the Poser Criteria [19] up to 2002 before being superseded by the McDonald Criteria [20–22]. Each patient’s MS course was defined as relapsing or progressive at onset. Demographic and clinical data including successive EDSS scores, relapses and use of disease modifying drugs (DMD) were assessed for each patient. DMD treatments were classified as: immunosuppressants (including cyclophosphamide, mitoxantrone, fingolimod, natalizumab, azathioprine, methotrexate and mycophenolate mofetil), immunomodulatory drugs (including interferons-beta, glatiramer acetate, dimethylfumarate and teriflunomide), never treated and unknown.

Disability was assessed by certified neurologists using the Kurtzke EDSS [5]. We used in statistical analysis the EDMUS impairment scale-disability status scale (EIS-DSS), which is an evaluation of the irreversible EDSS score since the previous visit (6 months– 1 year) translated into whole values [18]. The term “EDSS”, rather than “EIS-DSS”, is used in this paper for reasons of simplification.

EDSS 3 and 6 were considered as the most relevant scores during the course of the disease, as proposed by Leray and colleagues [23].

### Definitions of origin

Ethnic origin is reported in our database and was used to identify patients of European or of NA origin (i.e., from Morocco, Algeria, or Tunisia). We used the place of birth to determine NAG1 and NAG2: NA patients born in Morocco, Algeria or Tunisia were considered to be NAG1; NA patients born in France were classified as NAG2. Patients were excluded if the origin was different from Europeans or NA, or unknown. We collect no information about ethnicity of the parents of patients, leaving the option of patients of mixed origin (NA and Europeans).

### Statistical analysis

NAG1 and NAG2 patients were compared with European patients as reference with False Discovery Rate adjustment for multiple comparisons. Continuous variables were expressed by means (standard deviation SD), compared with ANOVA, and categorical variables by numbers and percentages compared by chi-square tests. Results were considered significant for a *P*<.05. Due to the small sample sizes, Fisher’s exact tests were used for comparisons of first DMD between groups.

Survival analysis were used to assess time to reach EDSS scores 3 and 6 from MS onset (origin date). Descriptions were performed by Kaplan-Meier estimates (median time, 95% confidence interval 95% CI) on the total follow-up. Patients who had already reached EDSS 3 (or more) at onset were excluded for the estimate of time to reach EDSS 3, and those who had a score of 6 or more were excluded of both estimates. Failure curves were reduced to the first 15 years from MS onset due to very small sample sizes thereafter.

To test the effects of patient characteristics on time to endpoints, Cox proportional hazard regressions were used for the first 15 years of follow-up. The explanatory variable of interest was the “origin/generation”, classified into three categories: NAG1/NAG2/Europeans (Europeans as reference). Models were adjusted for variables considered as potential confounders in the literature [24]: age at disease onset, gender, type of MS at onset (relapsing or progressive), EDSS at onset (<2 or ≥2) and annualized relapse rate (ARR) during the first 5 years, with log-transformation if log-linearity was not verified. The proportional hazards assumption was also tested for each covariate using log(-log(survival)) plot, Schoenfeld residuals and time-dependent covariate test method. Covariates were introduced in the multivariate model if associated with the explanatory variables during the bivariate analysis with *P*<.2. Hazard ratios (HR) and their 95% CI for disability were calculated and were considered significant if *P*<.05.

The data analysis for this work was performed by MS and generated using SAS software, Version 9.4 of the SAS System for Windows. (Copyright (c) 2017 by SAS Institute Inc., Cary, NC, USA).

## Results

We identified 5,502 MS patients collected between January 1^st^, 1996 and February 1^st^, 2016. Of these, 5,200 were Europeans with a mean follow-up (SD) of 15.6 years (y) (10.7), 275 were NA with a mean follow-up of 11.8 y (8.2), and 27 were excluded because of other or unknown origin. Among the NA patients: 80 were NAG1 with a mean follow-up of 12.9 y (8.1), and 167 NAG2 with a mean follow-up of 11.2 y (8.3). Generation information was missing for 28 patients, they were excluded from the analysis.

### Demographic and clinical data

The main demographic and clinical findings are shown in Table 1.

**Table 1:** Demographic and clinical data of patients. Europeans, North African (NA) of first generation (NAG1) and second generation (NAG2).

	**Europeans** n = 5200	**NA**		
		**NA** n = 275	**NAG1** n = 80	**NAG2** n = 167
**Sex: female, No (%)**	3750 (72.1)	178 (64.7)	44 (55.0)	119 (71.3)
		.008*	.001*	.81*
**Age at onset, mean (SD), years**	33.4 (10.7)	30.0 (9.7)	32.1 (9.5)	29.0 (9.3)
		*<*.001*	*0*.29*	*<*.001*
**Calendar year of the first symptoms, mean (SD)**	1996 (11.2)	2000 (8.5)	1999 (8.7)	2001 (8.4)
		*<*.001*	.01*	*<*.001*
**MS type at onset:**				
**Relapsing at onset, No (%)**	4538 (87.3)	245 (89.1)	66 (82.5)	154 (92.2)
**Progressive at onset, No (%)**	662 (12.7)	30 (10.9)	14 (17.5)	13 (7.8)
		.38*	.*21* *	.12*
**Complete recovery after the first relapse (EDSS <2), No (%)**	3821 (73.5)	212 (77,1)	54 (67.5)	137 (82.0)
		.19*	.23*	.03*
**Annualized relapse rate during the first 5 years, mean (SD)**	0.6 (0.4)	0.6 (0.4)	0.6 (0.4)	0.6 (0.4)
		.85*	.70*	.70*
**Time between the two first relapses, mean (SD), years**	3.7 (4.8)	3.7 (5.1)	3.6 (5.6)	3.7 (5.2)
		.97*	.96*	.96*
**Time from MS first symptoms to the beginning of first DMD, mean (SD), years**	6.0 (7.0)	5.2 (6.1)	5.0 (5.6)	5.3 (6.3)
		.07*	.26*	.26*
	**Europeans** n = 5200	**NA**		
		**NA** n = 275	**NAG1** n = 80	**NAG2** n = 167
**Sex: female, No (%)**	3750 (72.1)	178 (64.7)	44 (55.0)	119 (71.3)
		.008*	.001*	.81*
**Age at onset, mean (SD), years**	33.4 (10.7)	30.0 (9.7)	32.1 (9.5)	29.0 (9.3)
		*<*.001*	*0*.29*	*<*.001*
**Calendar year of the first symptoms, mean (SD)**	1996 (11.2)	2000 (8.5)	1999 (8.7)	2001 (8.4)
		*<*.001*	.01*	*<*.001*
**MS type at onset:**				
**Relapsing at onset, No (%)**	4538 (87.3)	245 (89.1)	66 (82.5)	154 (92.2)
**Progressive at onset, No (%)**	662 (12.7)	30 (10.9)	14 (17.5)	13 (7.8)
		.38*	.*21* *	.12*
**Complete recovery after the first relapse (EDSS <2), No (%)**	3821 (73.5)	212 (77,1)	54 (67.5)	137 (82.0)
		.19*	.23*	.03*
**Annualized relapse rate during the first 5 years, mean (SD)**	0.6 (0.4)	0.6 (0.4)	0.6 (0.4)	0.6 (0.4)
		.85*	.70*	.70*
**Time between the two first relapses, mean (SD), years**	3.7 (4.8)	3.7 (5.1)	3.6 (5.6)	3.7 (5.2)
		.97*	.96*	.96*
**Time from MS first symptoms to the beginning of first DMD, mean (SD), years**	6.0 (7.0)	5.2 (6.1)	5.0 (5.6)	5.3 (6.3)
		.07*	.26*	.26*

* Bivariate False Discovery Rate adjusted comparisons with E as reference (ANOVA for continuous variables; chi-square tests for categorical variables).

*P* in bold if<.05.

MS: multiple sclerosis; SD: standard deviation; DMD: disease modifying drug.

The female to male ratio (F:M ratio) was close to parity among the NAG1, but much higher (approximately 3:1) in Europeans and NAG2. First symptoms occurred 5 years earlier in NAG2 patients than in European patients and they recovered more frequently than Europeans from their first relapse.

We also studied the clinical symptoms of the first relapse. More NAG1 patients had sexual problems (8.8% *vs* 3.3% in E; *P* = .03) and oculomotor disorders were more frequent in NAG2 patients (20.4% *vs* 11.0% in Europeans, *P*<.001). No differences in mono/multisymptomatic first relapse were found.

The first DMD was different between NAG2 and European patients (*P*<.001): NAG2 patients were less likely to have used immunosuppressants as first DMD (10.8% *vs* 22.9% in Europeans). No significant difference in first DMD was found between NAG1 and European patients.

### EDSS 3 and EDSS 6

**Fig 1:**
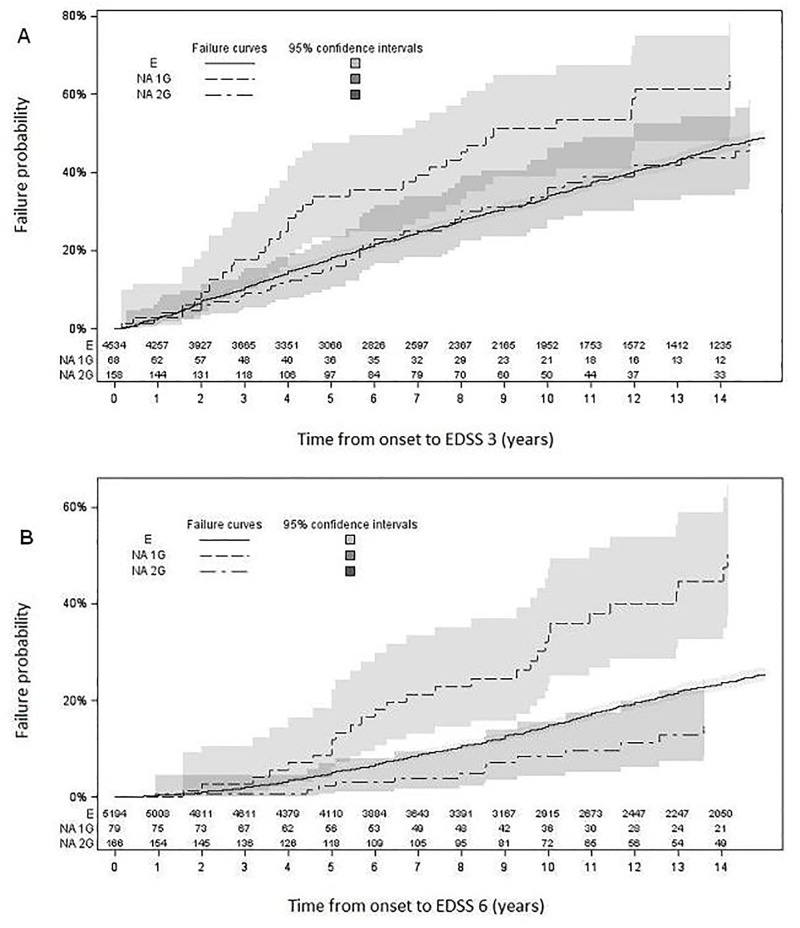
Survival curves for expanded disability status scale (EDSS) scores 3 (A) and 6 (B) of patients. First generation North Africans (NA1G), second generation North Africans (NA2G) and Europeans (E).

Median time estimates from MS onset to EDSS 3 were 13 y (95% CI 12–13) for European patients, 7 y (95% CI 4–12) for NAG1 and 15 y (95% CI 10–16) for NAG2 (Fig 1A).

Median times from disease onset to EDSS 6 were 26 y (95% CI 25–27) for European patients, 14 y (95% CI 10–36) for NAG1, and 39 y (95% CI 23–39) for NAG2 (Fig 1B).

Cox proportional hazard models are reported in Table 2.

**Table 2:** Association between “origin/generation” and assignment to expanded disability status scale (EDSS) scores 3 and 6.

	Hazard ratio	95% confidence interval	*P*
**EDSS 3**			
**NAG1 (ref: Europeans)**	**1.74**	**1.24–2.44**	**.002**
**NAG2 (ref: Europeans)**	**1.16**	**0.87–1.54**	.33
Male (ref: female)	1.37	1.23–1.51	**<.0001**
Age at onset (for each one year)	1.04	1.03–1.04	**<.0001**
Relapsing at onset MS (ref: progressive at onset MS)	*0*.*28*	0.23–0.33	**<.0001**
EDSS 2 or more at onset (ref: ≥2)	2.57	2.29–2.88	**<.0001**
ARR during the first five years (LOG) (for each log unit)	2.15	2–2.32	**<.0001**
**EDSS 6**			
**NAG1 (ref: Europeans)**	**2.38**	**1.63–3.47**	**<0.0001**
**NAG2 (ref: Europeans)**	**0.81**	**0.47–1.40**	.45
Male (ref: female)	1.28	1.12–1.46	**.0003**
Age at onset (for each one year)	1.03	1.03–1.04	**<0.0001**
Relapsing at onset MS (ref: progressive at onset MS)	*0*.*21*	0.17–0.26	**<0.0001**
EDSS 2 or more at onset (ref: ≥2)	2.59	2.19–3.06	**<0.0001**
ARR during the first five years (LOG) (for each log unit)	2.25	2.01–2.51	**<0.0001**

Adjusted Cox proportional hazard regression models. North Africans of first generation (NAG1); North Africans of second generation (NAG2); Europeans.

*P* in bold if <.05.

MS: multiple sclerosis; ARR: annualized relapse rate.

Relative to Europeans, persons in the NAG1 cohort had a higher risk of reaching EDSS 3 and 6, while the NAG2 cohort had no altered risk following adjustment for covariates.

## Discussion

We found two different phenotypes among NA MS patients in France depending on their generation: NA patients born in France have a similar disability progression profile to that of patients of European origin, whereas those born in their country of origin have a higher risk of advanced EDSS scores than Europeans.

NAG1 patients had a similar age at disease onset, but lower F:M ratio than the Europeans MS patients, while the NAG2 cohort had an earlier disease onset, but similar F:M to the Europeans. It is not surprising to observe a high F:M ratio among NAG2 if they are considered as having a more “European” phenotype, as an increasing F:M ratio is one of the most striking features of MS in Northern countries [25]. Concerning age at onset, this discrepancy between NAG2 and Europeans and NAG1 might be explained by the artificial lack of late onset in the NAG2 cohort (with a calendar year slightly more recent than NAG1 and significantly different from Europeans). A “migration bias” may also have influenced the differential F:M ratio and age at onset between NAG1 and NAG2. Migrants of the first generation were probably more likely to be healthy male workers; women and men having begun their disease in late childhood may have been poor candidates for migration.

The severity of the first relapse was assessed by the proportion of patients with full recovery. In the bivariate analysis, NAG2 patients were more likely to achieve full recovery than Europeans, while there was no significant difference between the NAG1 and European patients. This may be related to the younger age at first relapse in the NAG2 cohort, as recovery rates are generally better among younger patients [26].

With these baseline differences taken into account by the Cox model, we found that NAG1 patients were characterised by a higher risk of reaching EDSS 3 and 6 relative to Europeans, while NAG2 had comparable rates. This could be due to differences in environmental exposures between NAG1 and NAG2 as they play a key role in the risk of developing MS [27]. Other studies have also considered environmental exposures and the risk of disability progression [28], such as the effect of cigarette smoking or the average serum vitamin D levels in the first 12 months following a clinically isolated syndrome. Unfortunately we had no information about vitamin D status, smoking habits or other environmental exposures in our database and can’t further hypothesize. Further, we cannot rule out that modifications in genetics partly explain these results. Some of the NAG2 patients might have received European alleles in an unknown proportion, as we had no information about the origin of the parents of the NAG2 patients. A recent epidemiological study in France reported that up to 44% of second generation NA immigrants came from mixed union, i.e., with only one parent born in North Africa [29]. In the previously mentioned Norwegian study that introduced the concept of a “generational effect” on the incidence of MS in immigrants in Norway [13], immigrants of the second generation had both parents of foreign origin and nevertheless had a different risk of MS than immigrants of the first generation, which is in favour of the role of the environment in MS features. Another challenging question is the possible effect of socioeconomic status on medical practice and MS [8,30]. The socioeconomic conditions are certainly lower for a recently arrived immigrant (NAG1), than for the following generations. We were unable to consider the effect of socioeconomic status, as we do not collect this information.

One recent French study assessed the phenotype of MS among NA living in France, NA living in Tunisia and French patients of European origin [31]. They highlighted a difference in progressing to EDSS 6 between NA and European patients, and found significantly shorter times to reach EDSS 3, 4 and 6 in NAG2 patients living in France in comparison with Europeans. This was not the case between NAG1 patients living in France, NA living in Tunisia and Europeans. These discrepancies with our results might be the consequence of small sample size, and of a biased NAG2 cohort of patients with more aggressive MS, unadjusted on predictive factors such as the early annualized relapse rate (the log-rank test used to compare times to outcomes does not adjust on these “baseline” differences).

Our study has some limitations. First, we can’t insure that every Europeans and NA patients are collected. A previous capture-recapture study on our registry about its three major sources (neurologists declaration, the Health Insurance systems and the French Hospital Information System database) indicated that 10.1% of prevalent MS cases in Lorraine might be missing [32]. Moreover, this selection bias may be unbalanced between NAG1, NAG2 and Europeans, as first generation immigrants might use health services differently than persons that are established in a country. Thus, less severe cases might be more frequently missing in the NAG1 cohort. This, in addition with the “migration bias” as stated above may make our NAG1 group not representative of every MS patients born in North Africa. Younger cases may be missing, as they probably didn’t migrate in Europe. Unfortunately we didn’t know the date of migration or age at migration of our NAG1 patients, nor do we know whether some of them had their MS onset prior to their arrival in France. While Cox models were adjusted for age at MS onset, we did not have information on date of MS diagnosis, therefore could not determine if diagnostic delay may have played a role in some of these differences.

Eventually, the attrition bias seems to be limited since mean follow-up of NAG1 (12.9 y (8.1)) and NAG2 (11.2 y (8.3)) are quite similar.

## Conclusions

Our study suggests that there is a generational effect on MS phenotype among persons from North Africa who immigrated to France. The worse prognosis for persons born in North Africa was not reflected in the following generations of persons of NA ethnicity born in France, who had a similar disability trajectories as persons of European origin. To further evaluate the challenging question of the influence of environment and genetics in MS, further studies should control for parents’ origin in second generation NA immigrants, and for socioeconomic status and factors of environment exposure.

## Supporting information

S1 Fig Survival curves for expanded disability status scale (EDSS) scores 3 (A) and 6 (B) of patients. First generation North Africans (NA1G), second generation North Africans (NA2G) and Europeans.
MS: multiple sclerosis.
(TIF)
