# Supplementary figures and images for: Generational changes in multiple sclerosis phenotype in North African immigrants in France: A population-based observational study
Source: PLoS One. 2018 Mar 27;13(3):e0194115. doi: 10.1371/journal.pone.0194115 (PMC5870962; doi:10.1371/journal.pone.0194115)

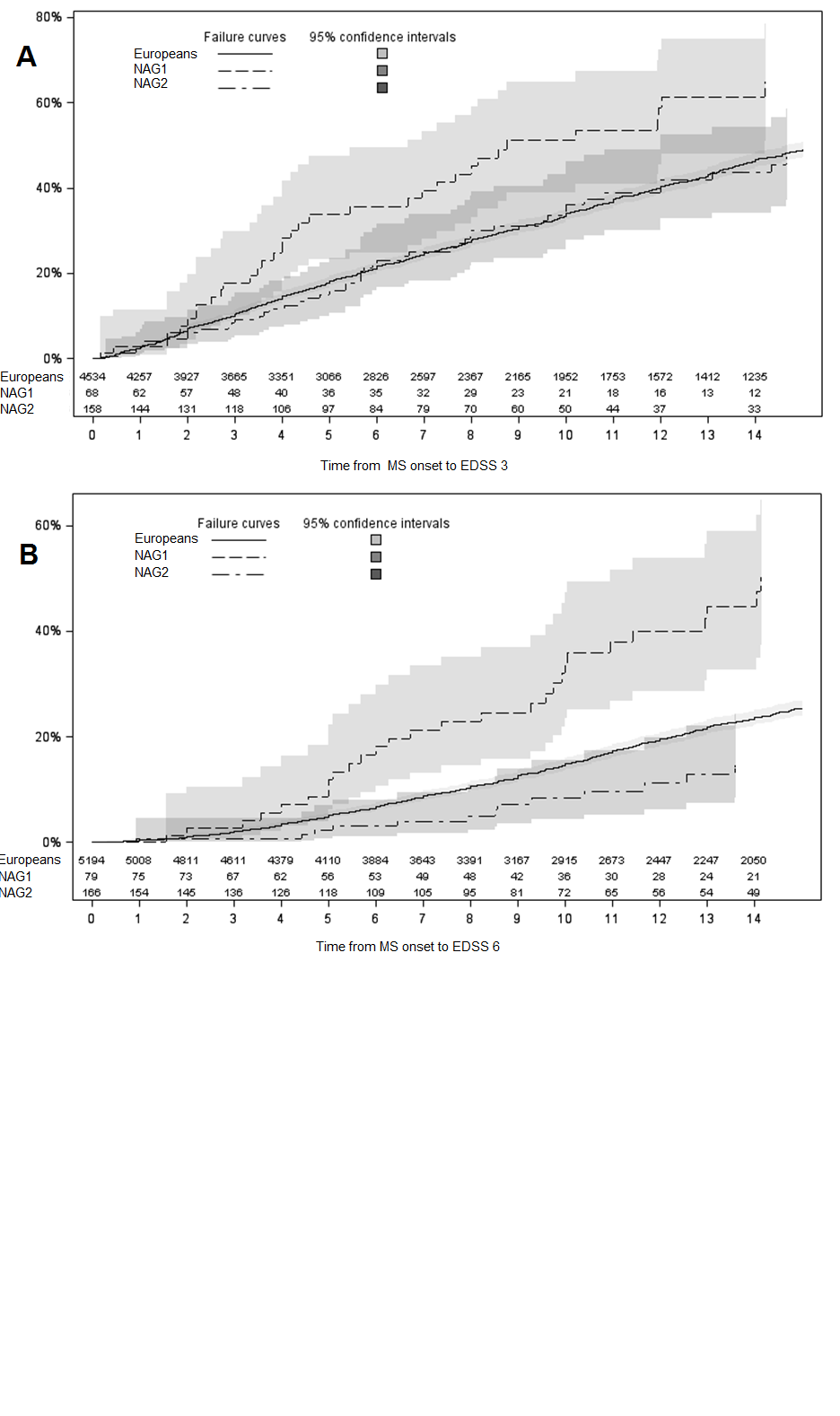

Supplement: S1 Fig — First generation North Africans (NA1G), second generation North Africans (NA2G) and Europeans. MS: multiple sclerosis. (TIF) [file pone.0194115.s001.tif]
